# Update on vasculitis: overview and relevant dermatological aspects for the clinical and histopathological diagnosis – Part II^☆^^☆☆^

**DOI:** 10.1016/j.abd.2020.04.004

**Published:** 2020-05-24

**Authors:** Thâmara Cristiane Alves Batista Morita, Paulo Ricardo Criado, Roberta Fachini Jardim Criado, Gabriela Franco S. Trés, Mirian Nacagami Sotto

**Affiliations:** a Department of Dermatology, Hospital das Clínicas, Faculdade de Medicina, Universidade de São Paulo, São Paulo, SP, Brazil; b Dermatology Discipline, Faculdade de Medicina do ABC, Santo André, SP, Brazil

**Keywords:** Anti-neutrophil cytoplasmic antibodies, Churg-Strauss syndrome, Henoch-Schönlein purple, Leukocytoclastic cutaneous vasculitis, Systemic vasculitis, Vasculitis, Vasculitis associated with lupus of the central nervous system

## Abstract

Vasculitis is a group of several clinical conditions in which the main histopathological finding is fibrinoid necrosis in the walls of blood vessels. This article assesses the main dermatological aspects relevant to the clinical and laboratory diagnosis of small- and medium-vessel cutaneous and systemic vasculitis syndromes. The most important aspects of treatment are also discussed.

## Clinical spectrum of vasculitis

In daily clinical practice, dermatologists are often asked to assess two groups of patients: those who present with small vessel vasculitis, and those who present with cutaneous arteritis (involvement of medium-caliber vessels). In light of this scenario, the dermatological manifestations should be characterized at the time of the initial presentation, and the findings of an adequate biopsy (one that represents all affected skin layers) should be taken into account in order to reach an adequate diagnosis. The moment when the test is performed is also crucial, as it can be non-diagnostic when performed too early or too late. A recent lesion, within 24–48 h of onset, is the most suitable for a histopathological assessment. Lesions older than 48 h can present, regardless of the underlying vasculitis, a lymphocyte-rich infiltrate. The assessment of additional data, such as those provided by direct immunofluorescence (DIF), as well as the status of the anti-neutrophil cytoplasmic antibody (ANCA), will further narrow the differential diagnoses. DIF biopsy should be performed on a lesion within 8–24 h of onset. Otherwise, leukocytes degrade the immune complexes, leading to a false negative result.^1^, ^2^, ^3^, ^4^

### Cutaneous single organ small vessel vasculitis

**Figure 1:**
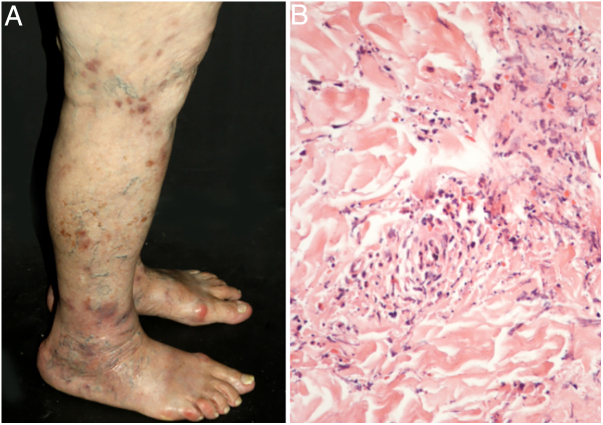
Cutaneous small vessel vasculitis limited to the skin: (a) palpable purpura and necrotic ulcers in the lower limbs; (b) necrosis of endothelial cells from superficial papillary dermis with fibrin deposition, neutrophil infiltration, and leukocytoclasia.

Cutaneous single organ small vessel vasculitis is a syndrome characterized by clinical and histological manifestations of cutaneous small vessel vasculitis (CSVV) without involvement of vessels of any other organ, which can be confirmed by anamnesis, physical examination, and specialized laboratory tests.^5^ Most cases manifest as palpable purpura or erythematous macules. However, hives, hemorrhagic vesicles, and shallow ulcers may be observed (Fig. 1).^2^, ^3^ Half of the patients present lesions exclusively on the lower limbs; however, the upper limbs, trunk, and gluteal region may also be involved.^6^ The disease is usually mild and self-limiting, with a good prognosis. Many patients with single-organ CSVV experience a single episode, which usually resolves within a few weeks. Recurrences are observed in about 10–25% of cases. In general, systemic symptoms such as fever, malaise, weight loss, and arthralgia may be observed in all cutaneous vasculitis syndromes.^7^, ^8^, ^9^

The classic histological findings of single-organ CSVV include a predominantly neutrophilic infiltrate affecting post-capillary venules, fibrinoid deposits, endothelial edema, and extravasation of red blood cells. A mixed inflammatory infiltrate can be observed, particularly in older lesions.^1^, ^3^ It is important to note that this histological pattern is not exclusive to any particular disease. Infections, insect bites, pyoderma gangrenosum, and even biopsies obtained from the bottom of chronic ulcers may exhibit the findings of leukocytoclastic vasculitis.^2^ Whenever possible, a second biopsy should be performed for DIF, whose findings reveal predominantly complement (C3), IgM, and fibrin deposits. Again, these findings are typical, but not specific, of any single-organ CSVV. Thus, the clinical–pathological correlation is mandatory before a definitive diagnosis is established.^4^

### Localized cutaneous vasculitis: erythema elevatum diutinum and facial granuloma

Erythema elevatum diutinum (EED) and granuloma faciale (GF) are forms of localized chronic cutaneous vasculitis that affect small-caliber dermal vessels. In EED, the lesions appear as well-defined papules, plaques or nodules, with an erythematous to brownish color, smooth surface, and relatively symmetrical distribution, with a chronic course and lasting around five to ten years. They occur preferably on the extensor surfaces of the limbs, a highly characteristic topography, especially on the skin over the joints. Rarely, EED can present as widespread papules on the upper and lower limbs, verrucous eruptions, or extensive nodular and fibrotic lesions on the palms and soles. Lesions may disappear spontaneously, without leaving scars, but atrophic hyper- or hypopigmented areas can be observed. This vasculitis most commonly affects female patients in any age group, although it is more frequently observed between the fourth and sixth decades of life.^10^, ^11^, ^12^

While the pathophysiology of EED is not fully understood, it is believed that skin lesions are caused by the deposition of immune complexes in small dermal vessels, which results in complement fixation and subsequent inflammation. Several publications relate EED to hematological abnormalities, particularly monoclonal gammopathies due to IgA; infections, by the human immunodeficiency virus (HIV), hepatitis C (HCV), hepatitis B (HBV), or tuberculosis; autoimmune diseases, such as rheumatoid arthritis, recurrent polychondritis, and inflammatory bowel disease; and solid lung or breast tumors.^13^ IgA-class ANCA may be a relevant marker of the disease. Immunoelectrophoresis should be used to investigate possible associated gammopathies.^11^, ^12^ EED can progress with ophthalmologic alterations, including nodular scleritis, panuveitis, and peripheral keratitis.^14^

GF has a predilection for middle-aged individuals, especially males, and is characterized by the appearance of purplish to brownish, asymptomatic or slightly pruritic papules, plaques, or nodules, usually located on the face. It has a smooth surface and can enhance the follicular ostia. Eosinophilic angiocentric fibrosis is a variant that affects the nasal mucosa, and rarely coexists with facial skin lesions. Extrafacial involvement is exceptional, but it can be observed in photoexposed areas or in the genitalia. Although usually refractory to treatment and showing rare spontaneous resolution, GF has not been associated with systemic diseases. Its differential diagnoses include sarcoidosis, lymphomas and cutaneous pseudolymphomas, discoid lupus, leprosy, granulomatous rosacea, and Jessner lymphocytic infiltrate.^15^, ^16^

EED and GF are fibrous vasculitis without signs of granulomatous reaction. The appearance of old lesions is of “onion skin” concentric fibrosis around the vessels.^11^ Histologically, in GF there is a dense, superficial, and deep dermal polymorphic inflammatory infiltrate, mostly perivascular. Characteristically, an infiltrate composed of all types of inflammatory cells, including neutrophils, lymphocytes, plasmocytes, and eosinophils, is separated from the overlying epidermis by a narrow Grenz zone. In turn, in EED there is no specific histological finding that differentiates it from other leukocytoclastic vasculitis. The epidermis is not always spared, and the Grenz zone is not observed.^11^, ^14^ The vesicobullous, hemorrhagic, ulcerative, and annular forms of EED are unusual. IgA deposits at the level of the vesicles are detected in vesicobullous EED, a variant whose most relevant differential diagnosis is dermatitis herpetiformis.^17^

### Urticarial vasculitis

Urticarial vasculitis (UV) is characterized by recurrent episodes of urticaria lasting over 24–36 h with histopathological features of leukocytoclastic vasculitis, involving mainly post-capillary venules.^18^ This vasculitis has an increased prevalence in female patients, peaking in the fourth decade of life, being extremely rare in children.^19^ UV can be idiopathic (more often) or be associated with drug reactions and chronic diseases, such as infections, malignant neoplasias, hematological diseases, and connective tissue diseases, particularly systemic lupus erythematosus (SLE) and Sjögren's syndrome. Unlike chronic idiopathic urticaria, UV lesions progress with pain, tenderness, and local heat sensation; they present a red-to-brown-colored central region on diascopy, and may progress with residual hyperpigmentation. Other skin lesions, such as purpura or necrosis, are occasionally observed.^18^, ^19^ It is not restricted to the lower limbs: any body region may be affected.^20^ Angioedema is observed in less than half of the patients. Most cases present a benign chronic course lasting three to four years, responding poorly to antihistamines.^19^

UV is essentially divided into: (i) normocomplementemic (80% of cases), a mild, self-limited disorder with predominant skin involvement; (ii) hypocomplementemic urticarial vasculitis syndrome, a rare and potentially fatal condition with hypocomplementemia persisting for at least six months. This presentation is generally associated with systemic manifestations, such as arthritis and Jaccoud arthralgia/arthropathy, as well as with proteinuria and hematuria due to glomerulonephritis in up to 50% of patients; chronic obstructive pulmonary disease, the leading cause of death among patients with UV; conjunctivitis, uveitis, or episcleritis; gastrointestinal symptoms; and cardiovascular disease.^21^, ^22^

The clinical suspicion of UV occurs in cases of urticarial lesions with an atypical presentation and suggestive laboratory tests, such as the following: high erythrocyte sedimentation rate (ESR), observed in 42.6% of patients; positivity for antinuclear antibodies (ANA), in 33.4% of patients; low levels of C3, C4, and CH50, in approximately one-third of patients, especially in the most severe forms of the disease; and, the presence of suppressed anti-C1q and/or C1q antibodies in 55% of patients with hypocomplementemic urticarial vasculitis syndrome. However, the diagnosis must be confirmed through a skin biopsy. Low levels of C1q can also be found in SLE (61%), rheumatoid arthritis (20%), scleroderma (15%), Sjögren's syndrome (15%), mixed connective tissue disease (15%), and chronic infection by HCV (38%).^15^, ^19^, ^23^ Hypocomplementemic UV syndrome is clinically and immunologically similar to SLE, being considered by some authors to be a syndrome associated with SLE.^24^

### IgA vasculitis

IgA vasculitis (IgAV) is a small vessel systemic leukocytoclastic vasculitis, which affects arterioles, capillaries and, above all, venules; it is associated with (isolated or predominant) IgA deposition. Vasculitis is uncommon in the pediatric age group, with the exception of Kawasaki disease and IgAV, which accounts for over half of all cases of vasculitis in children; it is notably more common between 3 and 15 years of age. IgAV often occurs about ten days after an acute inflammatory condition. For a long time, group B streptococcal infections were considered the only causal factor associated with IgAV. However, it is now known that they account for less than one-third of all cases. IgAV can be triggered by a wide variety of infectious agents, such as viruses, bacteria, and perhaps protozoa, as well as hymenopteran bites, drugs, and immunization.^7^ Approximately 25% of cases occur in adults, with similar findings to those seen in children. However, although adults rarely present intestinal intussusception, they are at a higher risk of significant kidney involvement. Particularly in this age group, IgAV can occur during the course or before the diagnosis of malignant neoplasias.^6^, ^7^

An eruption that symmetrically affects the lower extremities and the gluteal region is an unmistakable sign of IgAV in children. Less severe injuries can affect the face, trunk, forearms, and wrists. They usually have well-defined margins, vary in size from a few millimeters to a few centimeters, do not disappear on diascopy, and typically occur in flares. They can also coalesce, forming large areas of skin involvement. Purpuric lesions usually disappear within a month, progressing to brownish hyperpigmentation for another two weeks. Localized and non-depressible edema is observed in approximately 50% of IgAV cases. It is usually painless and involves more than one area, usually the dorsal region of the hands, ankles, and feet. Less frequently, the periorbital tissues, lips, forehead, and scalp are affected.^7^

Several other manifestations and atypical findings can be observed in IgAV. They include Köbnerization in the lower limbs, inguinal region, abdominal waist, wrists, or forearms; Rumpel-Leede capillary-fragility phenomenon, which consists of the appearance of palpable purpura after the application of a blood pressure cuff on the arm, at an intermediate point between systolic pressure and diastolic pressure, for 5–10 min; and uni- or bilateral scrotal pouch involvement, characterized by erythema, edema, and moderate local sensitivity. Vesicles and blisters can be observed within two weeks after the onset of the first skin lesions. In 5–10% of cases, cutaneous findings persist for over two months. Likewise, recurrences, defined by the reappearance of dermatological manifestations after a period of four or more weeks of complete recovery, are observed in 5–10% of patients.^7^ Strict follow-up with urinary sediment analysis and blood pressure monitoring should be maintained for at least six months, considering that renal involvement in IgAV is typically mild and asymptomatic.^4^

In addition to urinary sediment analysis, the initial laboratory evaluation should include the blood urea, creatinine, and electrolytes, as well as complete blood count, coagulogram, and ESR. As with Kawasaki disease and many other inflammatory conditions, reactive thrombocytosis can be observed. Complement levels are usually normal, and ANA and ANCA are usually negative. The DIF finding of IgA deposits in small vessels is sensitive, but not specific to IgAV, since it can be detected in vasculitis associated with systemic diseases, such as SLE, cryoglobulinemia, paraproteinemia/gamopathy, erythema nodosum, venous stasis, vasculopathies secondary to coagulopathies, and livedoid vasculopathy.^4^, ^25^ To date, no treatment has been shown to reduce disease duration.^4^ Some authors classify Finkelstein-Seidlmayer syndrome as a disease distinct from IgAV, given that the former presents isolated skin involvement and that IgA deposits are found in no more than one-third of the cases.^7^

Various classifications for the diagnosis of IgAV have been proposed over the years. In 2010, the European League Against Rheumatism (EULAR)/PRINTO/PRES criteria were published. Palpable non-thrombocytopenic purpura, often clustered and usually on the lower limbs, is a mandatory finding. In addition, affected patients must present at least one of the following: diffuse abdominal pain; arthritis or arthralgia; renal impairment (proteinuria >0.3 g/24 h or albumin/creatinine ratio >30 mmoL/mg in an urine sample in the morning; and/or hematuria >5 red blood cells per high power field or 2 [+] or more per dipstick or hematic cylinders in the urine sediment), and biopsy with typical leukocytoclastic vasculitis or proliferative glomerulonephritis, with a predominant deposit of IgA.^25^ The “CAAR” scale for cutaneous, abdominal, joint, and renal involvement ranges from 0 to 3, where 0 indicates absence; 1, mild; 2, moderate; and 3, severe involvement. The disease activity index can be calculated by adding the score corresponding to each of the four items (Table 1).^7^

**Table 1:** Assessment of involvement in IgA vasculitis, based on the “CAAR” classification for cutaneous, abdominal, joint and renal signs and symptoms.

Skin involvement	Absent: no skin lesions;
	Moderate: skin lesions located on (a) buttocks and lower limbs and (b) on the trunk or upper limbs;
	Severe: cutaneous lesions located on (a) the gluteal area and lower limbs, (b) trunk and (c) upper limbs.

Abdominal involvement	Absent: no symptoms, no findings;
	Mild: mild abdominal pain (induced by physical examination);
	Moderate: moderate abdominal pain (transient complaints referred by the patient);
	Severe: severe abdominal pain and/or melena and/or hematemesis and/or intussusception.

Joint involvement	Absent: no symptoms, no findings;
	Mild: symptoms or findings of joint involvement, without functional abnormalities;
	Moderate: symptoms and findings of joint involvement that cause moderate functional impairment (*e.g*., claudication);
	Severe: symptoms and findings that cause moderate functional loss (*e.g.*, inability to walk).

Renal involvement	Absent: normal urinary sediment exam.
	Mild: pathological hematuria, normal proteinuria (negative dipstick or [+]).
	Moderate: pathological hematuria, mild to moderate proteinuria (dipstick + to [++]).
	Severe: pathological hematuria, severe proteinuria (dipstick ≥[+++]).

The “CAAR” scale for cutaneous, abdominal, joint and renal involvement in IgA vasculitis ranges from 0 to 3, where 0 indicates absence; 1, mild; 2, moderate; and 3, severe involvement. Disease activity can be calculated by adding the four items.

### Recurrent cutaneous eosinophilic vasculitis

Recurrent cutaneous eosinophilic vasculitis is a rare cutaneous necrotizing vasculitis of small dermal vessels. Histopathological examination presents an almost exclusively eosinophilic inflammatory infiltrate, with few perivascular neutrophils. It can manifest as annular urticarial plaques, itchy purpuric papules, angioedema, or necrotic lesions. There are no manifestations suggestive of systemic disease. The disease follows a recurrent course and responds promptly to systemic corticosteroids.^26^

Cytotoxic proteins from eosinophil granules are deposited close to blood vessels, suggesting that these cells mediate vascular damage. Eosinophils release interleukin (IL)-5, C4, and platelet-activating factor, which can lead to increased vascular permeability. In DIF, no deposition of Ig along the vessel walls is observed. Recurrent cutaneous eosinophilic vasculitis is generally associated with peripheral blood eosinophilia, which is a common feature of many diseases, such as hypereosinophilic syndrome, eosinophilic cellulitis, eosinophilic granulomatosis with polyangiitis (EGPA), and eosinophilic fasciitis, although it is not always correlated with disease severity. Particularly in hypereosinophilic syndrome and eosinophilic cellulitis, infiltration of eosinophilis is observed around the dermal vessels, but true necrotizing vasculitis is not observed.^26^

### Acute hemorrhagic edema of infancy

Acute hemorrhagic edema of infancy (AHEI) has been described in the literature under a variety of synonyms, including “Henoch-Schönlein purpura of infancy,” “infantile erythema multiforme,” and “urticarial vasculitis of infancy.” Classically, it is characterized by the triad of palpable purpuric lesions, edema, and fever. Mucous membrane involvement is rare. This vasculitis is more frequent in males, with an age distribution that varies between 2 and 60 months, although 80% of cases occur in children aged 6–24 months. In almost 75% of cases, it is temporarily associated, within up to two weeks, with a variety of viral or bacterial infections and immunization. Although its etiology remains unknown, AHEI is believed to be mediated by immune complexes.^3^, ^27^

To date, no classification criteria have been proposed. The diagnosis is usually clinical, through five signs and symptoms: (i) age up to 2 years; (ii) typical skin lesions: target-shaped, predominantly affecting the face over the malar regions, ears, and limbs, sparing the trunk; edema of the face, ears, and extremities, without pruritus or scratches; (iii) preserved general condition, without cyanosis or pallor, without lower temperature in the limbs and with capillary refill time less than two seconds, absence of hypo- or hyperventilation; (iv) absence of joint or abdominal involvement; and (v) spontaneous recovery within two to three weeks.^3^

Visceral involvement is rare; however, arthritis, gastrointestinal bleeding, and scrotal edema are described. In doubtful cases, skin biopsy can be of great value for diagnosis. Typical histological findings are leukocytoclastic vasculitis, with or without fibrinoid necrosis. DIF can reveal deposits of C3, fibrinogen, and IgG and, less commonly, IgM or IgE. IgA deposition is observed in one-third of cases. Given its benign and self-limited course, with complete spontaneous remission in one to three weeks, and since AHEI usually does not present a good response to corticosteroids, most authors advocate a conservative approach, except for cases that evolve with severe inflammation, such as those with scrotal inflammation.^27^

## Mixed vasculitis, predominantly of small- and medium-vessels

### ANCA-associated vasculitis

**Figure 2:**
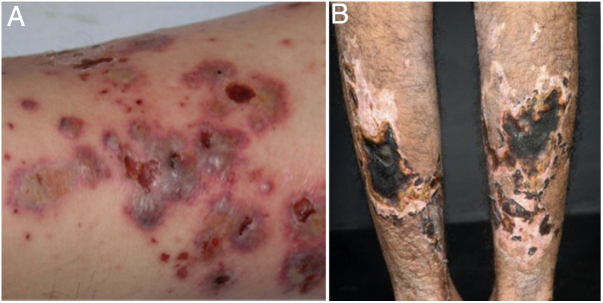
Anti-neutrophil cytoplasmic antibody (ANCA)-associated vasculitis: (a) eosinophilic granulomatosis with polyangiitis manifesting as palpable purpura and petechiae, both common and nonspecific manifestations of this group of vasculitis; (b) a patient with microscopic polyangiitis presenting bilateral necrotic ulcers on the lower limbs.

ANCA-associated vasculitis (AAV) may be limited to one organ, especially the lungs and kidneys, or may affect several systems from the initial presentation. Any type of vessel can be affected, including capillaries, venules, and arterioles. AAVs include EGPA, microscopic polyangiitis (MPA), granulomatosis with polyangiitis (GPA), and drug-induced AAVs; they exhibit a wide variety of skin manifestations, which can coexist in the same patient (Fig. 2). Therefore, diagnosis must be based on a detailed medical history, accurate physical examination, histopathological analysis of the skin biopsy, and ANCA status.^28^, ^29^, ^30^

Using indirect immunofluorescence (IIF), three main ANCA patterns have been described: cytoplasmic (c-ANCA), present in most patients with active GPA; perinuclear (p-ANCA), observed in patients with MPA and EGPA; and, atypical (a-ANCA), more rare. ANCAs may be related to exposure to drugs, particularly propylthiouracil, hydralazine, and cocaine. The presence and antigenic specificity of ANCAs provide potentially relevant clinical information: patients with c-ANCA or p-ANCA present impairment of different organ systems, as well as different patterns of response to induction drug therapy (prescribed for patients with the active form of the disease) and risk of recurrence. However, its simple presence does not confirm systemic vasculitis, considering that some cases of single-organ cutaneous vasculitis have positive ANCA, and that low levels of these autoantibodies can be found in several systemic inflammatory states and lung diseases.^28^, ^29^, ^30^

EGPA affects patients of both sexes, with a mean age at diagnosis of 50.3 ± 15.7 years. Asthma can precede systemic vasculitis by up to 20 years. The onset of sinusitis/nasal polyposis/rhinitis (58.8%); multiplex mononeuritis (46%); skin lesions (39.7%), especially palpable purpura, urticarial rash, and subcutaneous nodules typically located on the limbs and scalp, but also livedo and necrotic lesions; and pulmonary infiltrates (38.6%), reinforce EGPA suspicion. Cardiac, gastrointestinal, and renal involvement are observed in 27.4%, 23.2%, and 21.7% of patients, respectively.^31^, ^32^ Occasionally, the initial findings can be quite different from the usually recognized patterns, and this complexity can contribute to the delay in diagnosis and treatment. In the literature, the possible dermatological manifestations of EGPA vary from erythema multiforme-like to chronic lichenified lesions, previously diagnosed as nodular prurigo.^28^ Furthermore, although EGPA is a vasculitis associated with the presence of ANCA, approximately 50% of patients test negative for such autoantibodies.^32^

In MPA, skin lesions are found in 30–60% of patients and occur as an initial manifestation in 15–30% of cases. Palpable purpura are common (≥75%); however, the disease can also present with livedo, nodules, digital ischemia, and hives.^33^ EED-like nodules and plates have already been described. As in polyarteritis nodosa (PAN), dermatological manifestations are often associated with arthralgia and ophthalmologic involvement.^34^ Renal involvement is present in almost 80% of patients and is characterized by rapidly progressive glomerulonephritis. Regarding pulmonary involvement, the classic presentation is diffuse alveolar hemorrhage due to capillaritis^33^ In general, MPA evolves quickly, but there is a slowly progressive variant. The absence of findings such as microaneurysms and/or stenosis at angiography and positive p-ANCA (50–65% at the time of diagnosis) should be used as criteria for the diagnosis of MPA. However, some patients present positive c-ANCA.^33^

According to the 2012 Chapel Hill Conference Consensus (CHCC2012), GPA is defined as a necrotizing vasculitis of the skin, kidneys, and upper and lower respiratory tract.^35^ However, in the early stages, patients may experience only one or two of the triad's manifestations, and approximately 20% of cases may be c-ANCA negative.^36^, ^37^ Nasal sinus involvement is the most common finding and may be the only one observed in the localized form of the disease.^37^ Approximately 50–90% of patients present lung involvement, causing alveolar hemorrhage and/or parenchymal nodules. Skin lesions (10–50% of cases) are more frequent in those with severe and active multiorgan disease.^36^, ^37^ These lesions include palpable purpura; polymorphic lesions, including papules and necrotic nodules located on extensor surfaces, commonly on the elbows; pyoderma gangrenosum-like ulcers, also called malignant pyoderma; oral mucosa ulcers; gingival hyperplasia/strawberry gingivitis; and necrotic lesions of the penis.^36^, ^37^ Disease activity is correlated with ANCA levels. Mortality rate, as well as the frequency of recurrences, are high in all AAVs.^33^

Histologically, AAVs manifest as neutrophilic small vessel vasculitis. The c-ANCA/p-ANCA status assists in the interpretation of skin biopsy findings by the dermatopathologist. AAVs are often called “pauci-immune,” a term that refers to the absence of deposition of immune complexes in injured vessels. In the literature, EGPA and GPA, but not MPA, are classified as granulomatous vasculitis, a group that also comprises two large vessel vasculitis, giant cell arteritis (temporal), and Takayasu arteritis.^28^, ^29^, ^30^, ^34^, ^38^ In table 2, adapted from Marzano et al.,^28^ the cutaneous, systemic, and laboratory findings observed in AAV are summarized

**Table 2:** Findings in anti-neutrophil cytoplasmic antibody (ANCA)-associated vasculitis (antibodies to the neutrophil cytoplasm).

Organ/system findings	Granulomatosis with polyangiitis (GPA)	Eosinophilic granulomatosis with polyangiitis (EGPA)	Microscopic polyangiitis (MPA)
Cutaneous signs and symptoms	(10–50%) Palpable purpura, subcutaneous nodules, papules, vesicles, blisters, necrotic-ulcerative lesions, livedo reticularis/racemosa; pyoderma gangrenous-like ulcers. Ulceration and gangrene of the fingers or penis are rarely observed.	(40–50%) Subcutaneous nodules, purpura, livedo reticularis/racemosa, vesicles, aseptic pustules, hives, necrosis – ulcerative and maculopapular rash mimicking erythema multiforme with target lesions on the limbs, particularly on the palmar regions.	(30–60%) Palpable purpura, livedo reticularis/racemosa, nodules, hives lesions, skin ulcers with necrosis, blisters, erythema elevatum diutinum-like plaques. The limbs are most commonly affected and the lesions usually appear in an early stage of MPA. No relationship between any type of skin rash and incidence of renal and/or pulmonary involvement has been demonstrated.
Oral mucosa	(10–60%) Non-specific erosive/ulcerative oral lesions; strawberry gingivitis.	–	–
Upper and lower airways	(70–100%) Sinusitis with purulent or bloody discharge; ulceration of the nasal mucosa and palate; saddle nose deformity, perforation of the septum.	The prodromal or allergic phase is characterized by the occurrence of asthma (in approximately 95% of cases). Nasal polyposis, allergic rhinosinusitis, epistaxis.	Pulmonary involvement is frequent and can be observed in up to 90% of patients. The classic pulmonary manifestation is diffuse alveolar hemorrhage due to pulmonary capillaritis.
Kidney and urogenital tract	(40–100% of cases) Usually with a characteristic histopathology of pauci-immune focal necrotizing glomerulonephritis with crescents formation associated with extracapillary proliferation, which in turn can cause a wide range of clinical features, from urinary abnormalities to rapidly progressive renal failure. The severity of renal involvement remains the main prognostic factor for renal function, as well as for survival. Prostatitis, orchitis, epididymitis, ureteral stenosis.	In 25% of patients the most typical condition is a pauci-immune glomerulonephritis with crescents formation.	Renal involvement is present in almost 100% of patients and is characterized by rapidly progressive glomerulonephritis with a histological pattern of pauci-immune necrotizing glomerulonephritis with crescents formation.
Gastrointestinal system	Ulcerative lesions, intestinal perforation.	Eosinophilic gastroenteritis.	–
Peripheral nervous system	Multiplex mononeuritis, sensory-motor neuropathy.	Peripheral neuropathy.	Peripheral neuropathy.
Ophthalmologic involvement	Episcleritis, scleritis, corneal ulceration, retinal vasculitis, retro-orbital granulomatous pseudotumor, or dacryoadenitis.	–	–
Histopathological findings	Leukocytoclastic vasculitis with fibrinoid necrosis and neutrophilic infiltration of small dermal vessels; granulomatous inflammation around the vessels; nonspecific perivascular lymphocytic infiltration.	Leukocytoclastic vasculitis with fibrinoid necrosis and granulomatous inflammation involving venules; neutrophil-rich inflammatory infiltrate. Tissue eosinophilia.	Leukocytoclastic vasculitis with fibrinoid necrosis and neutrophilic infiltration of small dermal vessels; nonspecific perivascular infiltrate. A lymphocytic infiltration can be observed; cutaneous nodules often indicate vasculitis involving vessels in the deep dermis or subcutaneous tissue.
Deposits in direct immunofluorescence of the skin	IgM and/or C3 deposits around small dermal vessels in almost 70% of cases.	IgM and/or C3 deposits around small dermal vessels in over 50% of cases.	Usually negative; eventually IgM and/or C3 deposits are observed around small vessels.

C, complement; EED, erythema elevatum diutinum; EGPA, eosinophilic granulomatosis with polyangiitis; GPA, granulomatosis with polyangiitis; Ig, immunoglobulin; MPA, microscopic polyangiitis.

## Medium vessel vasculitis

### Systemic polyarteritis nodosa

Systemic polyarteritis nodosa (PAN) is a necrotizing vasculitis that typically affects medium-caliber muscular arteries.^39^ According to the definition proposed by the CHCC2012, in PAN there is no involvement of post-capillary venules.^35^ This entity has historically been divided into two main subtypes, systemic PAN and cutaneous PAN; the latter is now called cutaneous arteritis. The 1990 American College of Rheumatology (ACR) classification criteria for adult PAN and the EULAR classification criteria for infant PAN are summarized in table 3. Regarding skin manifestations, only livedo reticularis is considered a diagnostic criterion by the ACR, whereas all types of lesions were included by EULAR. Systemic PAN is a potentially fatal disease. In turn, cutaneous arteritis is a chronic benign condition, with a recurrent course. There is controversy in the literature regarding the possibility of progression from the exclusively cutaneous variant to the systemic form; if it occurs, it is probably unusual.^39^, ^40^, ^41^, ^42^

**Table 3:** Classification criteria for systemic polyarteritis nodosa in adults (1990 American College of Rheumatology) and for infantile polyarteritis nodosa (European League Against Rheumatism – EULAR).

1990 American College of Rheumatology (ACR) Classification criteria for adult PAN	Polyarteritis nodosa is diagnosed if at least three of these ten criteria are present. The presence of three or more criteria correlates with a sensitivity of 82.2% and a specificity of 86.6%.	1. Weight loss > 4 kg;
		2. Livedo reticularis;
		3. Testicular pain or tenderness (usually unilateral, secondary to vasculitis of the testicular artery, requiring emergency treatment due to the risk of irreversible ischemia);
		4. Myalgia, weakness or tenderness in the legs;
		5. Mononeuropathy or polyneuropathy;
		6. Diastolic BP > 90 mmHg;
		7. Elevated urea > 40 mg/dL or creatinine > 1.5 mg/dL, except for dehydration or obstruction;
		8. Presence of hepatitis B surface antigen or antibody in serum;
		9. Arteriogram showing aneurysms or visceral artery occlusions, except for arteriosclerosis, fibromuscular dysplasia, or other non-inflammatory causes;
		10. Small or medium artery biopsy containing PMN.

European League Against Rheumatism (EULAR) Classification criteria childhood PAN	A systemic illness accompanied by either a biopsy showing small and mid-size artery necrotizing vasculitis or angiographic abnormalities (aneurysms or occlusions) (mandatory criteria) (should include conventional angiography if magnetic resonance angiography is negative), plus at least two of the following seven criteria:	1. Skin involvement (livedo reticularis, tender subcutaneous nodules, other vasculitic lesions);
		2. Myalgia or muscle tenderness;
		3. Systemic hypertension, relative to childhood normative data;
		4. Mononeuropathy or polyneuropathy;
		5. Abnormal urine analysis and/or impaired renal function (glomerular filtration rate < 50% of normal for age);
		6. Testicular pain or tenderness;
		7. Signs or symptoms suggesting vasculitis of any other major organ system (gastrointestinal, cardiac, pulmonary, or central nervous system).

BP, blood pressure; PAN, polyarteritis nodosa; PMN, polymorphonuclear neutrophils.

PAN most frequently affects males and is observed in all ethnic groups. The mean age at onset of the disease is around 50 years, and the peak incidence occurs in the fifth to sixth decades of life.^34^, ^43^ The pathogenesis of PAN is not yet fully understood, although the clinical response to immunosuppressive therapy supports the concept that the mechanisms associated with the innate and adaptive immune systems play an active role in the disease. The main environmental factor associated with PAN is HBV infection. In these patients, the disease is more severe. Thus, the reduction in the prevalence of PAN cases in recent years may be related to the decrease in rates of HBV infection achieved by wide vaccination, blood transfusion screening, more effective treatment of the disease, and better recognition of other forms of systemic necrotizing vasculitis (*e.g*., AAV, cryoglobulinemic vasculitis).^39^, ^44^

Fever, weight loss, severe myalgia, and arthralgia affecting large joints are observed in over half of the patients.^39^ The disease spectrum ranges from single-organ involvement to polyvisceral failure, with a marked tendency to spare the lungs. Muscles and joints, peripheral nerves, and skin are the areas most frequently involved, although any organ can be affected.^43^ In most cases, neurological involvement presents as multiple mononeuropathy. However, asymmetric polyneuropathy and involvement of the central nervous system may also be observed. From 2% to 50% of patients with PAN present skin lesions, which may be purpura or hives, livedo racemosa, large ulcers, subcutaneous nodules and, more rarely, digital infarction or limb gangrene.^34^, ^44^

Renal involvement in PAN occurs at the level of the pre-glomerular arteries, causing malignant hypertension and/or renal failure, but without glomerulonephritis, which is a secondary manifestation of smaller vessel involvement. Abdominal and renal angiograms may indicate microaneurysms and/or stenosis.^34^, ^44^ Orchitis, included in the ACR classification criteria (Table 3) and detected in 24% of patients, is generally unilateral and secondary to testicular artery vasculitis.^43^ Abdominal pain occurs in one-third of patients. Digestive manifestations can be severe, with risk of hemorrhage and/or perforation, mainly in the small intestine. The main ophthalmological alterations are bilateral retinal detachment and retinal vasculitis.^39^

Histologically, vascular inflammatory lesions are segmental, mainly at the bifurcation points. In the acute phase, fibrinoid necrosis of the middle layer is observed, with an infiltrate that is initially composed mainly of polymorphonuclear neutrophils, and subsequently becomes predominantly lymphocytic/histiocytic. Aneurysms and thrombosis may complicate inflammatory vascular lesions. The healing phase is characterized by fibrous endarteritis that can lead to vascular occlusion. In a single biopsy sample, it is possible to observe the coexistence of lesions at different stages. No granulomatous inflammation is observed.^39^ ANCA, which is strongly correlated with small vessel involvement, is typically negative in PAN; however, Young et al.^43^ observed a rate of 19% positivity for these autoantibodies in 27 Korean patients who met the 1990 ACR criteria.

At diagnosis, a five-factor score (FFS) can be calculated to estimate the risk of mortality. These five factors consist of: proteinuria >1 g/day, renal failure (serum creatinine >1.58 mg/dL), gastrointestinal symptoms, cardiomyopathy, and central nervous system involvement.^43^ Among patients with an FFS of 2, the mortality rate is 33.3%. The first year of illness is a critical period for this outcome to occur.^33^

### Cutaneous arteritis

Cutaneous arteritis is a recurrent chronic necrotizing vasculitis that affects small arteries and arterioles in the deep dermis and/or hypodermis.^35^ In a retrospective review of 22 patients diagnosed with this vasculitis, the present study group found a predilection for white women, with a mean age of 39.4 years.^45^ Various infectious (group A beta-hemolytic *Streptococcus*, HBV, HCV, HIV, parvovirus B19, and *Mycobacterium tuberculosis*) and non-infectious conditions (autoimmune diseases, inflammatory bowel disease, neoplasms, immunization) have been reported in association with cutaneous arteritis. Drugs such as penicillin and tetracycline are also related to cutaneous arteritis, and minocycline is the main drug involved. In these cases, skin lesion improvement is observed when the medications are discontinued.^40^, ^46^, ^47^

Cutaneous arteritis is included in the group of immune complex-mediated vasculitis. DIF usually indicates deposits of IgM and C3 on the wall of the affected arteries.^44^, ^45^ Moreover, a 77.8% prevalence of IgM antibodies against the phosphatidylserine–prothrombin complex in patients with cutaneous arteritis corroborates the theory that prothrombin bound to apoptotic endothelial cells induces an immune response. This process would then lead to the development of anti-phosphatidylserine–prothrombin complex antibodies, which, in turn, activate the classic complement pathway, causing vascular damage.^44^

**Figure 3:**
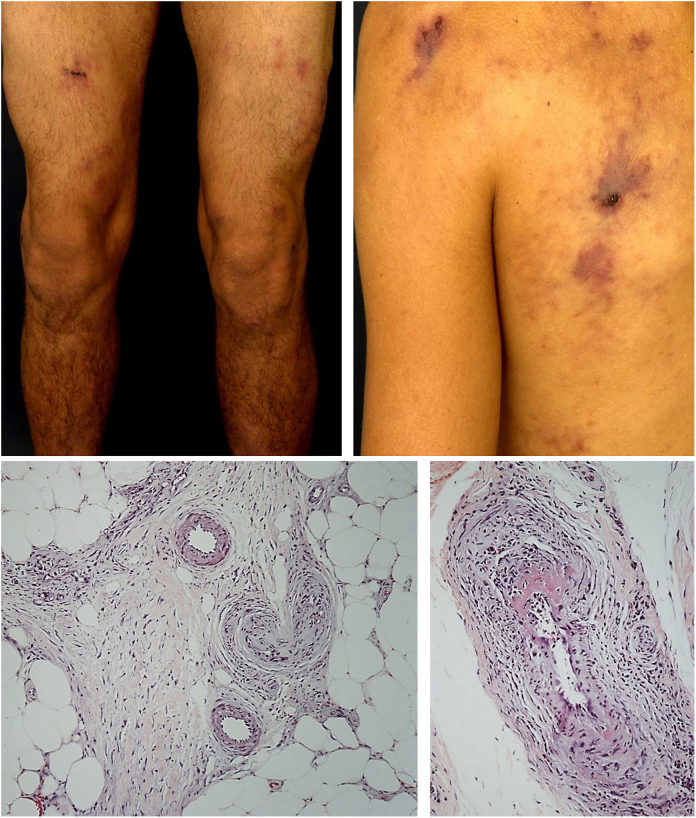
Cutaneous arteritis with retiform purpura in areas of previous livedo racemosa and histopathological examination, evidencing true vasculitis in the subcutaneous tissue.

The most frequent cutaneous manifestations are ulcers, livedo racemosa, and dermal or subcutaneous nodules, located mainly in the lower portion of the legs and the perimalleolar region, and that may ascend to the thighs and buttocks, occasionally affecting the hands and feet. The ulcers regress, leaving stellar atrophic scars, of ivory color, called atrophie blanche (Fig. 3), a manifestation that can be observed in occlusive vasculopathies such as livedoid vasculopathy. Peripheral neuropathy was identified in 25–32% of patients with cutaneous arteritis.^40^, ^45^ Although less common, extracutaneous findings also include malaise, fever, myalgia, and arthralgia.^44^, ^45^

In the cutaneous arteritis biopsy samples, Morimoto and Chen identified four stages: acute, subacute, reparative, and healing, according to the type of predominant inflammatory infiltrate and the morphological alterations observed in the vascular wall. They range from prominent neutrophil infiltration in the vessel wall to minimal cellular inflammation with vascular lumen occlusion by fibrin thrombi and neovascularization. Multiple stages can coexist in the same or in different biopsy samples from a patient.^48^

Nakamura et al.^40^ established the clinical and histological criteria to confirm the diagnosis of the disease. They are listed in table 4. A detailed assessment of autoimmune conditions, thrombophilia, and infectious diseases must be carried out. In countries where tuberculosis is endemic, the Mantoux test is particularly important.^45^

**Table 4:** Diagnostic criteria for cutaneous arteritis, previously known as cutaneous polyarteritis nodosa.

Compatible clinical findings	Subcutaneous nodules, livedo, purpura or ulcers.
Histopathological findings	Fibrinoid necrotizing vasculitis of small and medium caliber arteries.
Exclusion manifestation	Fever (≥38 °C, ≥2 weeks)
	Weight loss (≥6 kg in 6 months);
	Arterial hypertension;
	Rapidly progressive renal failure, renal infarction;
	Cerebral hemorrhage, cerebral infarction;
	Myocardial infarction, ischemic heart disease, pericarditis, heart failure;
	Pleuritis;
	Intestinal bleeding, intestinal infarction;
	Peripheral neuropathy outside the affected skin region;
	Arthralgia/arthritis or myalgia/myositis outside the affected skin region;
	Abnormal arteriography (multiple microaneurysms, stenosis, and obliteration).

**Figure 4:**
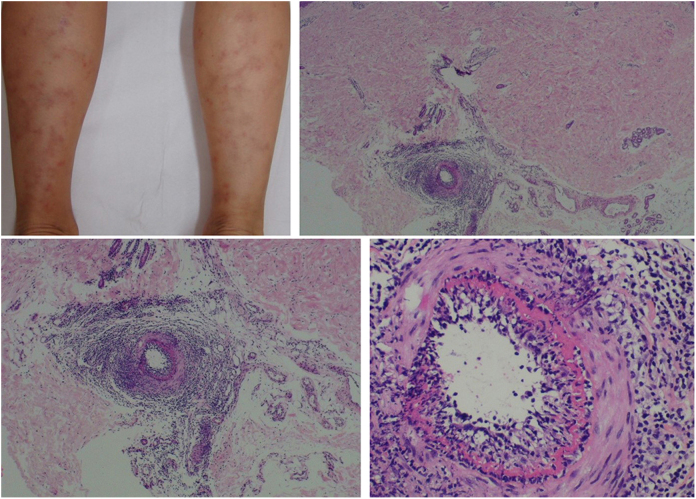
Macular lymphocytic arteritis (lymphocytic thrombophilic arteritis) showing livedo-type lesions without ulceration in the legs and the corresponding histopathological examination evidencing, at the dermo-hypodermic junction, an arterial vessel surrounded by lymphocytic infiltrate and with a fibrin ring in the intimal layer.

The clinical presentation of macular lymphocytic arteritis (MLA), also known as lymphocytic thrombophilic arteritis, includes hyperpigmented macules or plates and livedo racemosa, predominantly in the lower limbs. Although MLA is more commonly asymptomatic, there may be a propensity for neurological involvement, similar to other forms of cutaneous vasculitis. The pathogenesis of this entity is still unclear; the debate remains as to whether MLA is a new cutaneous vasculitis syndrome or an indolent variant of cutaneous arteritis. Histologically, it is characterized by a dense infiltrate of mononuclear cells in the muscle wall of the arterioles of the dermo-subcutaneous junction, with variable narrowing of its lumen by a typical hyalinized fibrin ring (Fig. 4). Contrary to the original description, during histopathological examination, rupture of the internal elastic lamina was observed in some patients.^49^, ^50^ Depending on the time of biopsy and the section examined, the subacute and reparative stages of cutaneous arteritis can be confused with MLA. Deep, repeated biopsies and serial cut analysis are sometimes necessary before concluding that there is no necrotizing arteritis, since the vessel involvement is segmental and focal.^42^, ^48^

### Adenosine deaminase 2 (ADA2) deficiency

Recent findings have attributed an autosomal recessive mutation in the CERC1 gene on chromosome 22q11 to one of the genetic defects associated with cutaneous arteritis. The CERC1 gene codes for adenosine deaminase 2 (ADA2), a group of enzymes involved in purine metabolism. ADA2 is synthesized only by the monocytic-macrophage system, cells with an important role in regulating the immune response. Several mutations that can be homozygous or compound heterozygotes have been detected, although their real prevalence worldwide remains unknown. Family history is negative in most cases, and the age at onset ranges from 2 months to 59 years.^51^, ^52^

ADA2 deficiency is an autoinflammatory syndrome that leads to several immune abnormalities, such as an increase in proinflammatory M1 macrophages (as opposed to anti-inflammatory M2 macrophages), which compromise endothelial integrity and establish a vicious circle of vasculopathy and inflammation. A significant proportion of children develop characteristics of B cell immunodeficiency, including recurrent infections, low levels of Ig, and cytopenia involving one or more strains. The clinical manifestations vary in severity, from limited skin involvement to severe multisystemic vasculitis with recurrent fever, renovascular hypertension/renal artery aneurysms, hepatosplenomegaly with portal hypertension, peripheral neuropathy, and stroke before the age of 5 years. Cutaneous findings include livedo racemosa, subcutaneous nodules, ulcerations, Raynaud's phenomenon, and finger necrosis in the most severe cases. In skin biopsies, non-granulomatous necrotizing vasculitis of medium-caliber arteries is usually observed; however, some patients may present leukocytoclastic vasculitis or even perivascular T lymphocytes, without clear vasculitis.^51^, ^52^, ^53^, ^54^

### Nodular vasculitis

**Figure 5:**
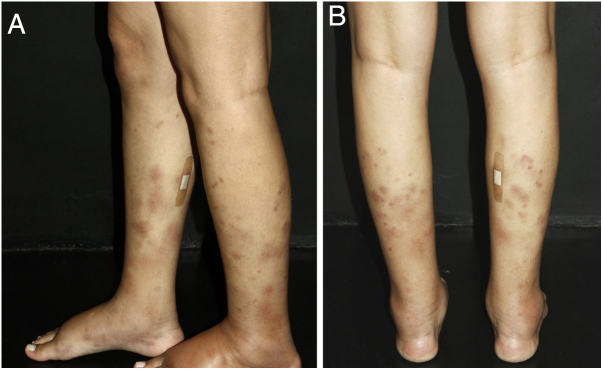
Nodular vasculitis: the posterolateral regions of the legs are affected by nodules and violaceous plaques. A bandage was placed where a skin biopsy had been done a few days earlier.

Nodular vasculitis (NV) is a predominantly lobular panniculitis, which presents with some type of vasculitis in the vast majority of cases. The age of the affected individuals ranges from 23 to 81 years (mean of 55.8 years).^55^ Typical patients are obese women with some degree of venous insufficiency, presenting episodes of nodules and plaques in the postero-lateral region of the lower limbs during winter months (Fig. 5). Injuries to the thighs, gluteals, and forearms may also occur. These areas are usually sensitive or painful at local pressure only, and often course with focal ulceration and drainage, causing scarring and post-inflammatory hyperpigmentation. The disease usually follows a chronic course, with episodes of acute exacerbation every three or four months. Patients are in good general health and do not present systemic signs and symptoms.^55^, ^56^, ^57^

For years, NV has been associated only with *M. tuberculosis* infection, as a tuberculoid-like manifestation. However, it has been described in patients with other infectious conditions, such as HBV, *Nocardia*, *Pseudomonas*, and *Fusarium*; non-infectious diseases, such as hypothyroidism, chronic lymphocytic leukemia, rheumatoid arthritis, and Crohn's disease; and possibly also as a medication-induced condition, since it has already been described during the treatment of psoriasis with etanercept, an inhibitor of the tumor necrosis factor (TNF)-α and in association with the use of propylthiouracil, with rapid resolution after is discontinuation.^55^

The most commonly found histopathological pattern is vasculitis involving small venules located in the center of the adipose lobe. Veins and (less frequently) septal arteries may be affected simultaneously or in isolation. In some cases, only obliterating endarteritis is observed. Extensive necrosis of the adipocytes of the lobules of the subcutaneous tissue may be identified. In fully developed lesions, a mixed infiltrate of epithelioid histiocytes, multinucleated giant cells, and lymphocytes contributes to the finding of lobular granulomatous panniculitis. Special stains do not demonstrate the presence of acid-alcohol resistant bacilli. The assessment of patients with suspected NV should include the search for fragments of *M. tuberculosis* DNA in tissue samples using PCR, chest radiography, tuberculin skin test, and serology for HBV and HCV. Evidence of active systemic tuberculosis is rare.^55^, ^56^

## Treatment of vasculitis

### Treatment of cutaneous vasculitis

In most cases, cutaneous vasculitis has a self-limiting course. Once systemic involvement is excluded, therapeutic procedures should focus on treating symptoms, preventing triggers (such as medication and prolonged exercise), and treating underlying infections. Rest, elevation of the lower limbs, and use of compression stockings may be useful to decrease the deposition of immune complexes related to stasis, and accelerate ulcer healing. There is no evidence of the benefit of topical corticosteroids in CSVV. Their only effect appears to be the reduction of the local inflammatory response, relieving the pruritus and the burning sensation. Often, non-steroidal anti-inflammatory drugs, mainly acetylsalicylic acid (1–3 g/day) and indomethacin (25–50 mg/three times a day) are sufficient, without the need to associate systemic corticosteroids.^4^, ^58^, ^59^

Therapeutic options for prolonged use include colchicine, which is particularly useful for skin and joint symptoms (0.5 mg, two to three times a day, dose limited by gastrointestinal side effects). Colchicine decreases neutrophilic degranulation and modifies the expression of endothelial adhesion molecules. Dapsone, whose anti-inflammatory effects occur through inhibition of neutrophils migration to areas of tissue damage, as well as inhibition of the activity of myeloperoxidase and lysosomal enzymes of the neutrophils, has proven efficacy when used alone or in combination with colchicine (initial dose 25–50 mg, titrated up to 200 mg/day). Dapsone is particularly useful in patients with EED. Its prescription requires screening for glucose-6-phosphate dehydrogenase deficiency and regular laboratory monitoring for hemolytic anemia, methemoglobinemia, and (less commonly) agranulocytosis. Other treatments for CSVV include hydroxychloroquine (200–400 mg/day), which is particularly beneficial in UV and in preventing vasculitis flares in patients with inactive SLE; and pentoxifylline (400–1200 mg/day), whose benefits from the combination with dapsone outweigh the results obtained with the use of either of these two drugs in isolation. H1 antihistamines (hydroxyzine 25 mg/day), alone or in combination with H2 antihistamines (ranitidine 150 mg/twice daily), can be used to relieve pruritus by blocking the release of histamine, in addition to decreasing vascular permeability to immune complexes.^4^, ^21^, ^58^

When none of these agents is effective or tolerated, and CSVV is significantly symptomatic and/or extensively ulcerative, systemic corticosteroids become the main therapeutic option (0.5–1 mg/kg/day of prednisone or prednisolone). For persistent/resistant cases, monotherapy with prednisone is not recommended, due to the possibility of adverse effects, especially in children; its use is then preferred in low doses as an adjuvant to treatment with corticosteroid-sparing agents, such as azathioprine, methotrexate, and mycophenolate mofetil. Cyclophosphamide and cyclosporine can be effective. Other specific treatments, such as the one for cryoglobulinemia, may include plasmapheresis to remove immune complexes.^58^, ^59^, ^60^

### Treatment of systemic vasculitis

Standard therapy for systemic vasculitis is initiated with high doses of oral corticosteroids (1–1.5 mg/kg/day up to 80 mg). When remission is achieved, decrease must be slow and progressive, in order to obtain a daily dose of prednisone in the range of 20 mg/day at three months, 10 mg/day at six months, and 5 mg/day at 12 months, until withdrawal between 12 and 24 months. Thus, as in CSVV, in severe or recurrent forms, corticosteroids should always be associated with immunosuppressive therapy. Cyclophosphamide may be administered orally (500 mg/day to 2 g/kg/day) or intravenously (600 mg/m^2^, every two to four weeks), which is generally preferred as it presents comparable efficacy with a lower rate of side effects. Once remission is achieved (three to six months), a switch to a maintenance regimen with methotrexate (15–25 mg/week), azathioprine (2 mg/kg/day), or cyclosporine (2.5–5.0 mg/kg/day administered orally, divided into two doses) is recommended in order to avoid possible complications of cyclophosphamide therapy. Oral or parenteral methotrexate can be used as a less toxic alternative to cyclophosphamide to induce remission in non-life-threatening AAVs or in cases without target organ damage. Mycophenolate mofetil (2 g/day orally) or leflunomide are used in patients who are intolerant or unresponsive to methotrexate or azathioprine.^3^, ^7^, ^30^

Treatment options for refractory cases include the anti-CD20 monoclonal antibody rituximab (500 mg every six months for 18 months) and TNF-α inhibitors (infliximab or etanercept), as well as intravenous immunoglobulin (200–1000 mg/kg/day). TNF-α inhibitors are useful in inducing remission of ADA2 deficiency, while other immunosuppressants did not achieve the expected results. Maintenance therapy for systemic vasculitis should be continued for 18–24 months after remission, due to the high frequency of relapses. In patients with PAN associated with HBV, conventional treatment regimens without antiviral therapy are contraindicated due to the risk of continuous viremia, progression of chronic hepatitis or cirrhosis and, more alarmingly, reactivation of the virus with fulminant hepatitis. Thus, a combination of corticosteroids, plasmapheresis, and antiviral therapy is recommended. Plasmapheresis is also used for patients with rapidly progressive severe kidney disease, in order to improve survival; in this scenario, it has proven to be superior to methylprednisolone pulses (1 g/day for three days). The treatment of the vasculitis described in this study is summarized in table 5. The posology of the listed drugs considers the dose for adult patients.

**Table 5:** Therapeutic approach for vasculitis according to the severity of the manifestations.

Clinical severity	Findings	Therapeutic approach
Mild vasculitis limited to the skin	Persistent or recurrent vasculitis and/or symptomatic diseases (burning or pruritus)	Dapsone or colchicine (useful for mild disease, IgAV, UV or Behçet's disease; both drugs have better therapeutic effects after two to four weeks);
		Dapsone 25–50 mg/day orally at the beginning of treatment. After a complete blood count and liver function laboratory evaluation with normal results, the dosage may be increased to 100–200 mg/day;
		Colchicine: 0.5 mg, two to three times daily, orally;
		Other agents: pentoxifylline 400 mg, three times daily (Behçet's disease or mild cutaneous arteritis).

Moderate to severe cutaneous vasculitis	Extensive skin involvement, persistent vasculitis, recurrent vasculitis with ulcers, nodules or recalcitrant symptoms	Methotrexate, azathioprine, and/or prednisone (cutaneous arteritis, mild vasculitis limited to the skin, rheumatoid vasculitis, lupus vasculitis);
		Methotrexate: 5–20 mg/week;
		Azathioprine (AZA; PNS involvement, renal involvement): TPMT (levels); TPMT < 5, not recommended; TPMT 5–13.7 U (AZA 0.5 mg/kg/day); TPMT 13.7–19 U (AZA 1.5 mg/kg/day); TPMT > 19 U (AZA 2.5 mg/kg/day).
		Prednisone: 1–1.5 mg/kg/day orally;
		Hydroxychloroquine: 400 mg/day orally;
		Cyclosporine 2.5–5.0 mg/kg/day orally;
		Cyclophosphamide (extensive skin and ulcerative vasculitis or PNS involvement, or renal involvement): 100 mg/day to 2000 mg/day, orally.

Systemic vasculitis	Potentially fatal disease or permanent organ damage, serum creatinine levels >150 μmoL/L (severe if >500 μmoL/L)	Prednisone ± cyclophosphamide or azathioprine or cyclosporine or mofetil mycophenolate (AAV, PAN, severe IgAV, lupus vasculitis, large vessel vasculitis);
		Methylprednisolone (intravenous) 1000 mg/day for three consecutive days, then prednisone 1.0–1.5 mg/kg/day orally;
		Mofetil mycophenolate: 2000 mg/day (HUV, AAV).

IgAV, IgA vasculitis; HUV, hypocomplementemic urticarial vasculitis; PAN, polyarteritis nodosa; TPMT, thiopurine S-methyltransferase; AAV, anti-neutrophil cytoplasmic antibody (ANCA)-associated vasculitis.

## Conclusion

In summary, the diagnosis and management of vasculitis must be multidisciplinary in most cases. In clinical practice, it is mandatory to assess the involvement of internal organs and systems as the first step for patients with skin lesions suggestive of vasculitis.

## Financial support

This work was carried out with the support of the Coordenação de Aperfeiçoamento de Pessoal de Nível Superior (CAPES) – Financing Code 00.

## Authors' contributions

Thâmara Cristiane Alves Batista Morita: Elaboration and writing of the manuscript.

Paulo Ricardo Criado: Approval of the final version of the manuscript; conception and planning of the study; elaboration and writing of the manuscript; effective participation in research orientation; intellectual participation in propaedeutic and/or therapeutic conduct of studied cases; critical review of the literature; critical review of the manuscript.

Roberta Fachini Jardim Criado: Approval of the final version of the manuscript; conception and planning of the study, elaboration and writing of the manuscript; effective participation in research orientation; critical review of the literature; critical review of the manuscript.

Gabriela Franco S. Trés: Approval of the final version of the manuscript; elaboration and writing of the manuscript; intellectual participation in propaedeutic and/or therapeutic conduct of studied cases; critical review of the literature; critical review of the manuscript.

Mirian Nacagami Sotto: Approval of the final version of the manuscript; intellectual participation in propaedeutic and/or therapeutic conduct of studied cases; critical review of the manuscript.

## Conflicts of interest

None declared.
